# The referral pattern and treatment modality for peri-implant disease between periodontists and non-periodontist dentists

**DOI:** 10.1186/s12903-023-03135-3

**Published:** 2023-06-27

**Authors:** Chia-Dan Cheng, Yi-Wen Cathy Tsai, Wan-Chien Cheng, Fu-Gong Lin, Pei-Wei Weng, Ying-Wu Chen, Ren-Yeong Huang, Wei-Liang Chen, Yi-Shing Shieh, Cheng-En Sung

**Affiliations:** 1 Department of Periodontology, School of Dentistry, Tri-Service General Hospital and National Defense Medical Center, Section 2, Chang-Gong Rd, Nei-Hu District, 114, No. 325 Taipei, Taiwan; 2 School of Public Health, National Defense Medical Center, Taipei, Taiwan; 3 Department of Optometry, Asia University, Taichung City, Taiwan; 4 Department of Orthopaedics, Shuang Ho Hospital, Taipei Medical University, New Taipei City, Taiwan; 5 Department of Orthopaedics, School of Medicine, College of Medicine, Taipei Medical University, Taipei, Taiwan; 6 Division of Geriatric Medicine, Department of Family Medicine, Tri-Service General Hospital, National Defense Medical Center, Taipei, Taiwan; 7 Department of Operative Dentistry and Endodontics, School of Dentistry, Tri-Service General Hospital and National Defense Medical Center, Taipei, Taiwan

**Keywords:** Referral, Treatment modality, Peri-implant disease, Periodontists, General practitioners

## Abstract

**Objectives:**

This study is to investigate the referral pattern and treatment modality of dentists in the management of peri-implant diseases between periodontists and non-periodontist dentists (NPDs).

**Materials and methods:**

A total of 167 validated questionnaires were obtained from periodontists and NPDs, who had experience of placing implants for at least one year. Question I to IV asked how the dentist would respond if a patient came for treatment of their peri-implant diseases with four different scenarios according to resource of patient and disease severity. For each Scenario, dentists also replied which treatment procedures they would use if they decide to treat the patient.

**Results:**

Periodontal training, resource of patient, and disease severity were shown to significantly influence the referral pattern and treatment modality in the management of peri-implant disease (*p* < 0.05). Periodontists were more likely to use variable treatment procedures, including occlusal adjustment (OR = 2.283, *p* < 0.01), oral hygiene instruction (OR = 3.751, *p* < 0.001), topical antiseptic agent (OR = 2.491, *p* < 0.005), non-surgical mechanical therapy (OR = 2.689, *p* < 0.001), surgical therapy (OR = 2.009, *p* < 0.01), and remove implant (OR = 3.486, *p* < 0.001) to treat peri-implant diseases, compared to NPDs.

**Conclusion:**

The periodontal specialty training, resource of patient, and disease severity significantly influenced the referral pattern and treatment modality of dentist treating an implant diagnosed with peri-implant disease. This study also highlighted the importance of educating basic periodontal and peri-implant disease-related knowledge to all dentists regularly performing dental implant treatments.

**Clinical relevance:**

Peri-implant diseases are highly prevalent among patients with dental implants. Periodontal specialty training could enhance using variable treatment procedures to treat peri-implant diseases for dentists.

**Supplementary Information:**

The online version contains supplementary material available at 10.1186/s12903-023-03135-3.

## Introduction

Dental implant therapy has been a well-established method to restore missing teeth [1]. Numerous studies have demonstrated that dental implants are able to increase patients’ chewing function, self-esteem, social life, and quality of life [2, 3]. A global cross-sectional study indicated that not only are general practitioners beginning to perform implant therapy, but also those within 0–2 years of graduation had a higher percentage of placing their first implants despite acknowledging they were not yet proficient in implant practice [4].

Although dental implant treatments have satisfied successful outcomes, pathogenesis of the peri-implant tissues can still present complications which threaten the long-term survival of implants, such as peri-implant diseases. Many studies have reported the varied prevalence of peri-implant mucositis [5]. according to different disease definitions. A study analyzing electronic oral health records in the educational institution showed even approximately 1/3 of the patients and 1/5 of all implants experienced peri-implantitis [6]. Compared to peri-implant mucositis, peri-implantitis is more difficult to manage for the problem of decontamination of the roughened and threaded surfaces on exposed implants, and required more advanced treatment modalities [7, 8].

With the demand to incorporate dental implants as a treatment option among dentists, treatment outcomes may vary depending on the education level or experience of the practitioner. A study had found the implant survival and success rates in general dental practices may be lower than those reported in studies conducted in academic or specialty settings [9]. The treatment planning and modality of implant therapy also varies greatly among dentists with different training backgrounds, or due to the complexity of diseases and medico-legal reasons [10–12]. Therefore, when more advanced implant-related surgical therapy was required, general dental practitioners might consider referring the patient to dental specialists or experienced dentists [13, 14].

To achieve a successful therapy for peri-implant disease, not only are accurate diagnosis and valid treatment procedures needed, but also proper referral and communication with the patients are demanded among general dentistry providers and specialists. Although many techniques and various researches for treatment of peri-implant disease were discussed, the study of referral pattern and treatment modality in dentists were limited [15]. Therefore, the aim of this study is to investigate the referral pattern and treatment modality in the management of peri-implant diseases between periodontists and non-periodontist dentists (NPDs).

## Materials and methods

### Ethics

This study had been approved by the human research ethics board of the institution (TSGHIRB No. 2–105-05–001) and was conducted in accordance with the Helsinki Declaration of 1975, as revised in 2013.

### Questionnaire interview

The hard-copy Chinese version of the questionnaires was distributed during three major academic annual conferences held by three academies in Taiwan: Academy of Family Dentistry (AFD), Taiwan Academy of Periodontology (TAP), and the Academy of Dental Implantology. Individuals participating in these three academic conferences were consecutively invited to be enrolled in this study. To ensure good comprehension and validity, a face-to-face interview was performed with each participant. The definition of peri-implant diseases was explained to all subjects according to the Consensus report of the 2017 World Workshop on the Classification of Periodontal and Peri-Implant Diseases [16]. The information page at the beginning of the questionnaire explicitly stated that participation was voluntary, and the informed consents for this study were also obtained from all subjects after completion of the questionnaire. The collected information for analysis, such as gender, age, years of practice (the period of actually engaging in clinical practice after graduation from the dental school), location of occupation, contact information, and certified specialties in order to differentiate their status as a periodontist or NPD, was confidential and anonymous.

Inclusion criteria include participants who:

- had a valid dental license in Taiwan;
- had the experience of implant placement in patients for more than one year;
- were actively practicing in Taiwan.

Exclusion criteria include participants who:

- did not complete the questionnaire;
- were retired, not actively practicing, or were practicing outside of Taiwan.

### Periodontal specialists and non-periodontist dentists (NPDs)

Periodontists had to be registered as an active member with the Taiwan Academy of Periodontology in Taiwan. According to the requirements for obtaining this board-certified license (http://www.twperio.org.tw/), specialists must have completed 24–36 months of full-time training in a board-accredited training institute. Clinically, they must become proficient in the diagnosis and treatment of periodontal disease, including the management of advanced cases. Once these requirements had been met, the student must pass the written and oral examination in the certification process to be qualified as a periodontal specialist. After qualification, they must reach the minimal requirements of 180 credits of continuing education courses every six years to maintain their status as specialists.

NPDs include other specialists or general practitioners with a valid dental license to practice in Taiwan. The process for acquiring a dental license includes obtaining a Doctor of Dental Surgery (DDS) degree by graduating from a dental education program accredited by the Ministry of Health and Welfare (https://www.mohw.gov.tw) and Ministry of Education, Taiwan, which usually consists of a 6-year education including 1 year of intern training, and the board-certified exam.

### Location of occupation

Four levels of medical facilities from Medical Center, Regional Hospital, District Hospital, and Local Clinic, were defined according to the Ministry of Health and Welfare in Taiwan (https://dep.mohw.gov.tw/DOMA/mp-106.html). In this study, the Regional Hospital and District Hospital were combined into the “Hospital” category. If the participants worked in either Medical Centers, Hospitals, or Local Clinics at the same time, they would be classified according to the order of Medical Center, Hospital, and Local Clinic.

### Design of the questionnaire

The questionnaire was designed by two of the investigators (Y.-W.C and C.-E.S.) and validated by three other periodontists (Y.-W.C.T., W.-C.C., and R.-Y.H.) working in the Periodontal subdivision*.

A six-part questionnaire was developed to obtain the treatment decision of each dentist on treatment of peri-implant diseases [16], which referred to previous studies investigating the diagnosis, treatment decision, and referral patterns of general dental practitioners and specialists [11, 12, 17–19] (Table S1).

Question I and II specifically ask how a dentist would respond when a patient diagnosed with peri-implant mucositis or peri-implantitis originally placed by another dentist came for treatment.

From Question I ~ II, the dentist had to answer by ranking the following treatment plan options:

1. Treat the patient by myself;
2. Refer the patient to a periodontal specialist;
3. Refer the patient to an oral surgeon;
4. Refer the patient to a dentist who has taken implant specialty courses;
5. Refer the patient to the original dentist.

Question III ~ IV, specifically ask how a dentist would respond when a patient diagnosed with peri-implant mucositis or peri-implantitis placed by him or her-self came for treatment.

For Question III ~ IV, the dentist had to answer by ranking the following treatment plan options:

1. Treat the patient by myself;
2. Refer the patient to a periodontal specialist;
3. Refer the patient to an oral surgeon;
4. Refer the patient to a dentist who has taken implant specialty courses.

In each Question I to IV, the dentists also had to choose which treatment procedure they would use if they decided to treat the patient as Scenario I to IV, including oral hygiene instruction, regular follow, systemic antibody application, topical antimicrobial agent, topical antibody application, non-surgical mechanical therapy, surgical therapy, remove implant, occlusal adjustment, and laser treatment.

Question V inquiry whether the dentist perceives that peri-implant diseases are associated with periodontitis as a method to assess his or her perception on the importance of periodontal management for peri-implant diseases. Question VI asked the dentist whether the dentist would like to become further educated in basic periodontology and peri-implant diseases in order to be able to manage such patients personally. For Questions V ~ VI, the dentist could simply tick the “YES” or “NO” box to complete answers.

### Reliability and validity

The Ƙ statistic for analysis was used to assess intra-rater reliability of this questionnaire by 20 randomly selected dentists before the study. The Ƙ statistic values for Question I to Question IV were 0.914, 0.898, 0.835, and 0.835, respectively. The validity of the study was assessed by retrospectively revisiting 30 respondents for their decision in the clinical practice after answering the questionnaires through their contact information. The sensitivity and specificity from Question I to Question IV were 85.71% and 100%, 80% and 100%, 100% and 83.3%, and 100% and 83.3%, respectively.

### Sample size calculation

Sample size was calculated based on the data obtained from a similar previous research studying decisions of dentists towards implant dentistry with respect to the dentists’ factors and their training factors [19]. The maximum likelihood function in logistic regression by square value to estimate sample size needed by using G*Power Version 3.1. The total minimum sample was 128 dental practitioners, including at least 64 periodontists and 64 NPDs required to achieve the power of 80% and OR of 0.4.

### Statistical analyses

The respondents’ demographics were grouped as either periodontists or NPDs. The comparison of treatment decisions between periodontists and NPDs from Question I to Question VI were analyzed by Chi-square tests. The treatment procedures selected by periodontists or NPDs in Scenario I to IV were also compared by Chi-square tests. The rank orders of “Answer 1. Treat the patient by myself” among Question I to Question IV were compared using Kruskal–Wallis H with post hoc tests (analysis of variance) to evaluate the inclination of the dentists for treating the patient between four different scenarios. Multivariable logistic regression with generalized estimating equation (GEE) method was used to investigate the factors influencing their first choice for referring when faced with peri-implant disease and their treatment modality if they decided to treat the patient. A significance level of 0.05 was used for all analyses performed by SPSS for Windows (Version 20.0 for Windows, SPSS, Inc., Chicago, IL, USA).

## Results

### Demographics of study subjects

A total of 167 questionnaires from 73 periodontists and 94 NPDs, were qualified for analysis. The demographics characteristics of subjects were shown in Table S2.

#### General responses from questions I-IV and scenario I-IV

**Fig. 1:**
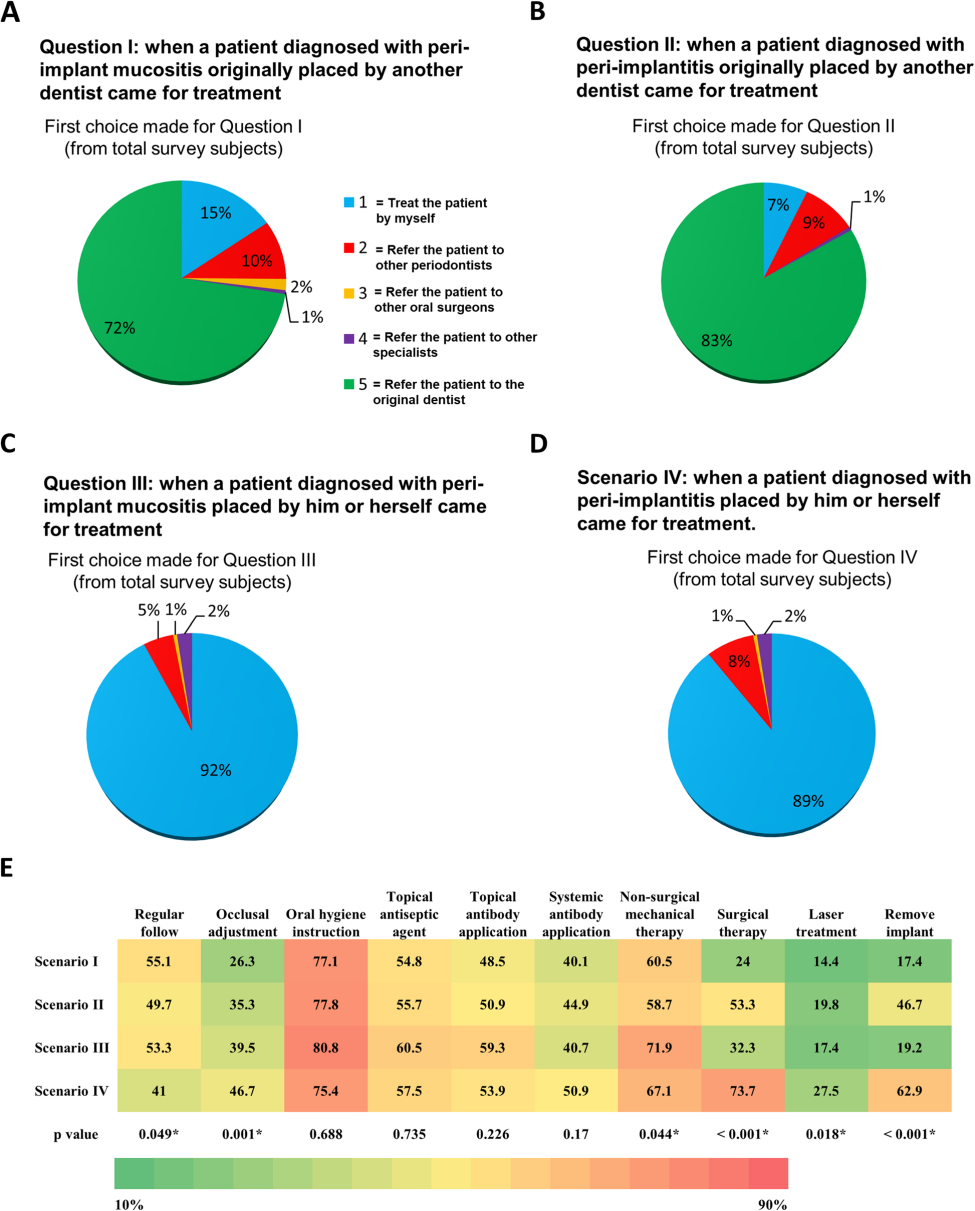
Summary of the treatment option with highest preference selected for Question I-IV and treatment procedures for Scenario I-IV by subjects (both NPDs and periodontists) in the questionnaires. 1: Treat the patient by myself; 2: Refer the patient to other periodontists; 3: Refer the patient to other oral surgeons; 4: Refer the patient to other implant specialists; 5: Refer the patient to the original dentist. **A** The subject’s preferred option for Question I of what to do when he/she encounters a patient with an implant diagnosed with peri-implant mucositis and the implant had been originally placed by another dentist. **B** The subject’s preferred option for Question II of what to do when he/she encounters a patient with an implant diagnosed with peri-implantitis and the implant had been originally placed by another dentist. **C** The subject’s preferred option for question III of what to do when he/she encounters a patient with an implant diagnosed with peri-implant mucositis and the implant had been originally placed by him/herself. **D** The subject’s preferred option for question IV of what to do when he/she encounters a patient with an implant diagnosed with peri-implantitis and the implant had been originally placed by him/herself. **E** The treatment procedures which subjects would use if they decide to treat the patient

The general response for Questions I-IV and Scenario I-IV were summarized (Fig. 1). When dentists faced a patient diagnosed with peri-implant mucositis or peri-implantitis originally placed by another dentist, the majority of responses (72% and 83%) were to refer the patient back to the original dentist as their first choice, respectively (Fig. 1A and B). As for the treatment decision of Question III and IV, when faced a patient diagnosed with peri-implant mucositis or peri-implantitis placed by him/herself, 92% and 89% of subjects preferred to treat the patient by themselves as their first choices, respectively (Fig. 1C and D). Oral hygiene instruction was the treatment procedures which most dentists would use for treating peri-implant disease (Fig. 1E). Different Scenarios could also affect the treatment procedure dentists used, especially for regular follow, occlusal adjustment, non-surgical mechanical therapy, surgical therapy, laser treatment, and remove implant (*p* < 0.05).

### Different scenarios according to disease severity and patient resource

In order to realize whether the disease severity and patient resource could influence the referral pattern, the ranking of the Answer 1, “treat the patient by myself” for Question I-IV, were analyzed from highest to lowest preference (Table 1). For Question I and II, most dentists ranked Answer 1 as the fifth preference, which represented 37.1% for peri-implant mucositis and 47.9% for peri-implantitis. Only 15.6% and 7.2% of dentists ranked Answer 1 as their first choice for Question I and II, respectively. However, 92.2% and 89.2% of dentists ranked Answer 1 as their first choice for Question III and IV, respectively. The distributions of the rankings for Answer 1 from Question I to IV were significantly different (*p* < 0.001).

**Table 1:** The ranking distribution of “Answer 1. Treat the patient by myself.” from Question I to Question IV

	First		Second		Third		Fourth		Fifth		Mean rank	p
	n	%	n	%	n	%	n	%	n	%		
Question** I**	26	15.6%	53	31.7%	17	10.2%	9	4.8%	62	37.1%	3.17^a^	< 0.001*
Question** II**	12	7.2%	49	29.3%	11	6.6%	15	9.0%	80	47.9%	3.61^b^	
Question** III**	154	92.2%	4	2.4%	0	0.0%	9	5.4%	0	0.0%	1.19^c^	
Question** IV**	149	89.2%	6	3.6%	1	0.6%	11	6.6%	0	0.0%	1.25^c^	

^*^: significant difference at *p* < 0.05 by Kruskal–Wallis H with post hoc tests

a, b and c designate significantly distinct data subsets with post-hoc analysis

### Comparison between periodontal specialists and NPDs

**Fig. 2:**
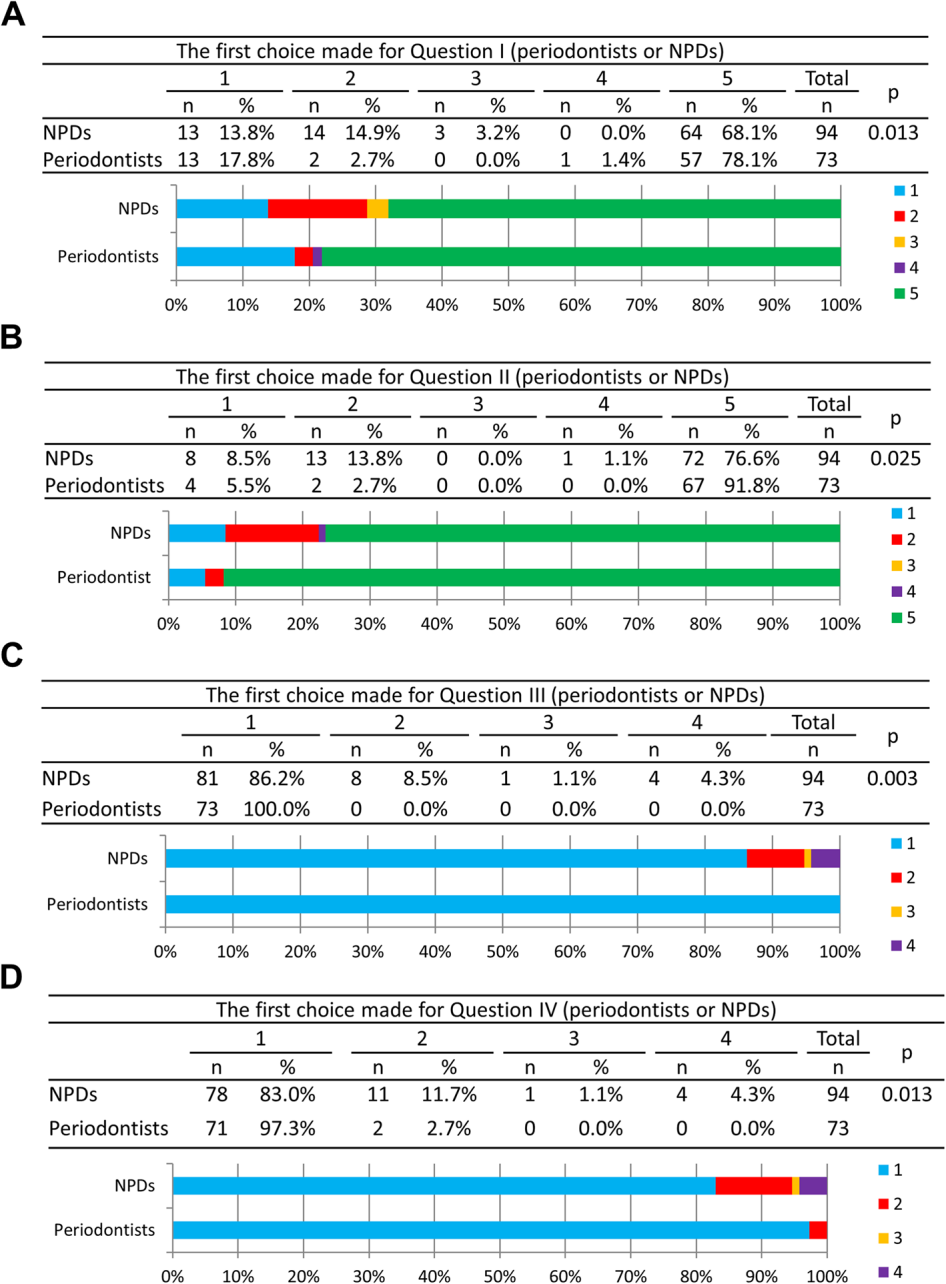
The first choice of all the treatment options selected is evaluated for periodontists and NPDs in response to Question I-IV. 1: Treat the patient by myself; 2: Refer the patient to other periodontists; 3: Refer the patient to other oral surgeons; 4: Refer the patient to other implant specialists; 5: Refer the patient to the original dentist. **A** The most preferred treatment option selected in response to Question I for periodontists and NPDs. **B** The most preferred treatment option selected in response to Question II for periodontists and NPDs. **C** The most preferred treatment option selected in response to Question III for periodontists and NPDs. **D** The most preferred treatment option selected in response to Question IV for periodontists and NPDs

**Fig. 3:**
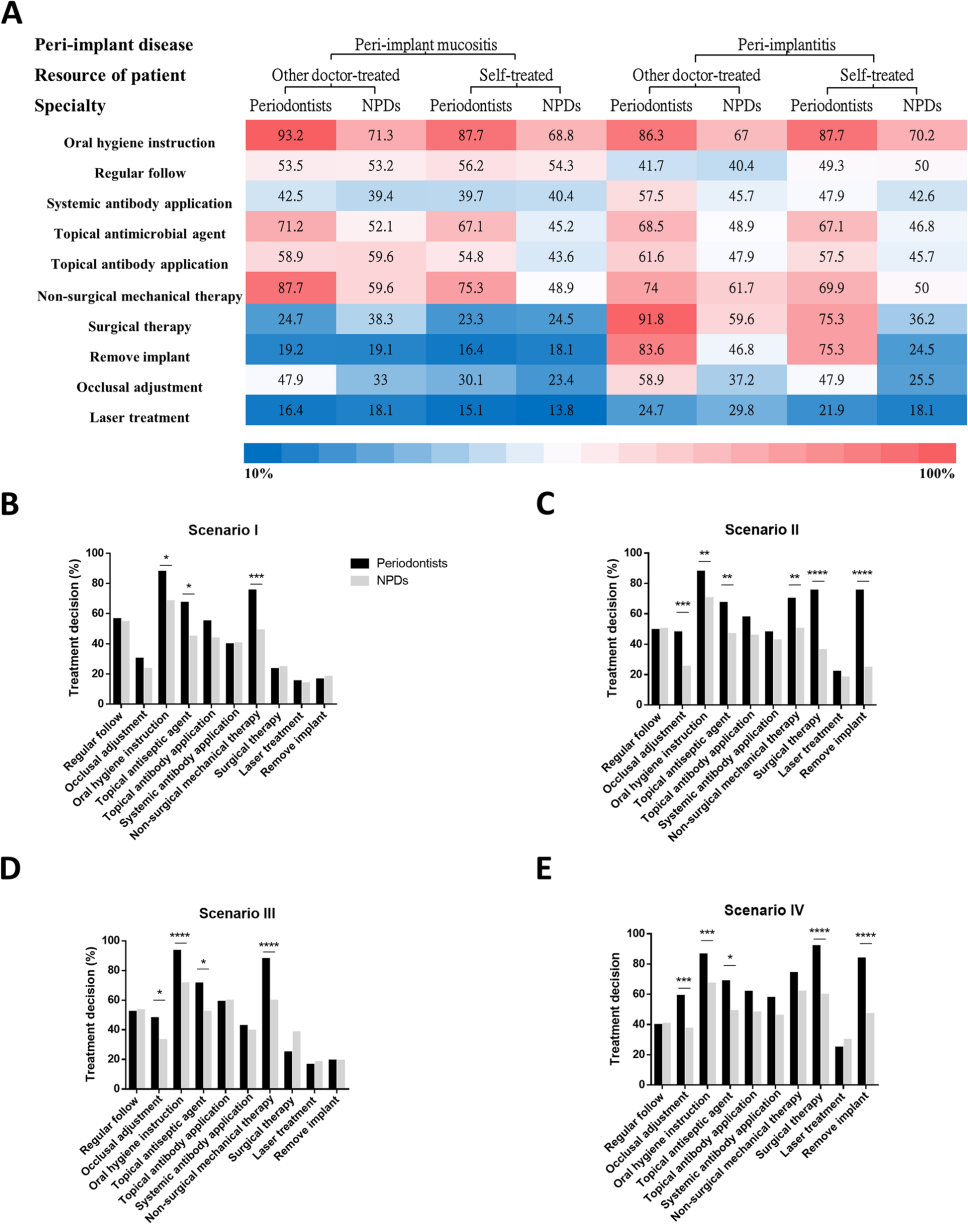
The treatment procedures which periodontists and NPDs would use in response to Scenario I-IV. **A** The overall selections by periodontists and NPDs in different Scenarios. **B** The treatment procedures selected by periodontists and NPDs in Scenario I. **C** The treatment procedures selected by periodontists and NPDs in Scenario II. **D** The treatment procedures selected by periodontists and NPDs in Scenario III. **E** The treatment procedures selected by periodontists and NPDs in Scenario IV. *, **, ***, and ***: significant difference at *p* < 0.05, *p* < 0.01, *p* < 0.005, and *p* < 0.05, respectively

The treatment decisions were compared between periodontists and NPDs for Question I to IV (Fig. 2 and Fig. 3). A significantly greater number of periodontists would choose to treat peri-implant mucositis by him/herself at 17.8% as their first choice in Question I, compared to NPDs at 13.8% (*p* = 0.013) (Fig. 2A). When faced with implants diagnosed with peri-implantitis and placed by another dentist (Question II), more NPDs (8.5%) were willing to treat the patient as their first choice, compared to periodontists (5.5%) (*p* = 0.025, Fig. 2B).

For Question III, 100% of periodontists, compared with only 86.2% of NPDs, would choose to treat peri-implant mucositis if the implant had been placed by him/herself (*p* = 0.003, Fig. 2C). The second highest ranked choice for NPDs was to refer the patient to a periodontal specialist at 8.5%. For Question IV when faced with peri-implantitis, 97.3% of periodontists would choose to treat the peri-implantitis, while only 83.0% of NPDs would choose to treat the diseased implant (*p* = 0.013, Fig. 2D**)**. The second highest ranked option choice for NPDs was to refer the patient to a periodontist at 11.7%.

A significantly greater number of periodontists would choose oral hygiene instruction, topical antiseptic agent, and non-surgical mechanical therapy in Scenario I (Fig. 3B); occlusal adjustment, oral hygiene instruction, topical antiseptic agent, non-surgical mechanical therapy, surgical therapy, and remove implant in Scenario II (Fig. 3C); occlusal adjustment, oral hygiene instruction, topical antiseptic agent, and non-surgical mechanical therapy in Scenario III (Fig. 3D); occlusal adjustment, oral hygiene instruction, topical antiseptic agent, surgical therapy, and remove implant in Scenario IV (Fig. 3E), to treat peri-implant disease (*p* < 0.05).

### Influence of years in practice on referral pattern and treatment modality

**Fig. 4:**
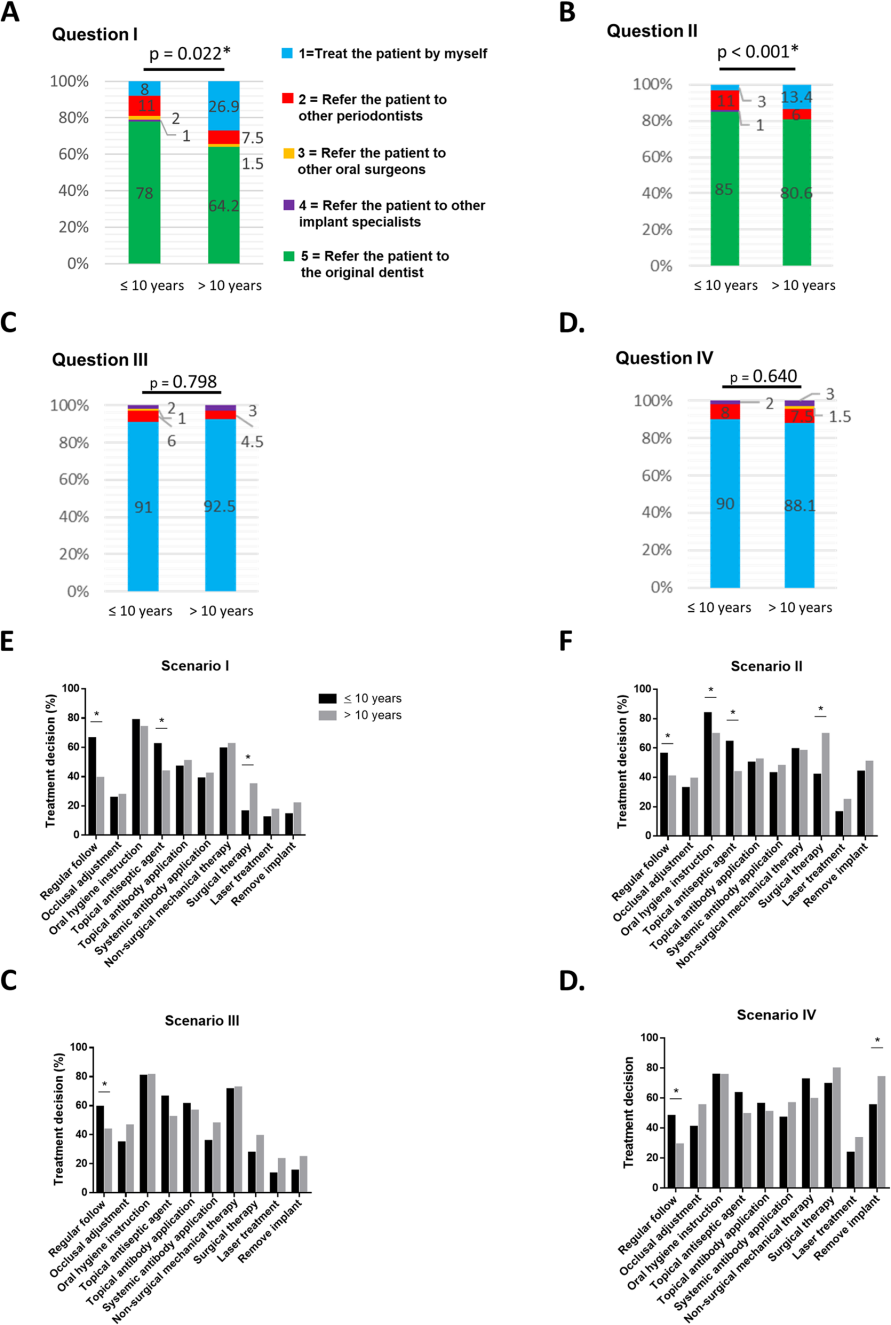
Difference of years in practice on referrals and treatment procedures by dentists with less than and equal to, or more than 10 years of clinical experience. 1: Treat the patient by myself; 2: Refer the patient to other periodontists; 3: Refer the patient to other oral surgeons; 4: Refer the patient to other implant specialists; 5: Refer the patient to the original dentist. **A** The subject’s preferred option for Question I of what to do when he/she encounters a patient with an implant diagnosed with peri-implant mucositis and the implant had been originally placed by another dentist. **B** The subject’s preferred option for Question II of what to do when he/she encounters a patient with an implant diagnosed with peri-implantitis and the implant had been originally placed by another dentist. **C** The subject’s preferred option for Question III of what to do when he/she encounters a patient with an implant diagnosed with peri-implant mucositis and the implant had been originally placed by him/herself. **D** The subject’s preferred option for Question IV of what to do when he/she encounters a patient with an implant diagnosed with peri-implantitis and the implant had been originally placed by him/herself. **E** The treatment procedures selected by dentists in Scenario I. **F** The treatment procedures selected by dentists in Scenario II. **G** The treatment procedures selected by dentists in Scenario III. **H** The treatment procedures selected by dentists in Scenario IV. *: significant difference at *p* < 0.05

The influence of dentists’ clinical experience on their decision for treating peri-implant diseases was summarized (Fig. 4). In Question I and II, dentists with > 10 years of clinical experience are significantly more likely to treat the implant disease themselves (*p* < 0.05). Moreover, dentists with > 10 years of clinical experience are significantly more likely to use surgical therapy in Scenario I and II, compared to dentists with ≤ 10 years of clinical experience (*p* < 0.05).

### Factors influencing the referral and treatment modality of dentists

In order to evaluate the factors which may influence whether a dentist would choose to refer a patient with peri-implant disease to other dentists or treat the peri-implant disease him/herself, and their treatment modality, multivariable logistic regression with the GEE method was performed (Tables 2 and 3). Dentists with a periodontal specialty (OR = 0.412, *p* = 0.004), or when faced with a patient’s peri-implant disease placed by him/herself (OR = 0.010, *p* < 0.001) were significantly more likely to consider treating the peri-implant disease him/herself. However, if the patient’s implant is diagnosed with peri-implantitis, subjects were more likely to refer the patient to another dentist for treatment (OR = 1.860, *p* < 0.001) (Table 2). When further analyzing which treatment procedure dentists would use, periodontists significantly preferred using occlusal adjustment (OR = 2.283, *p* < 0.01), oral hygiene instruction (OR = 3.751, *p* < 0.001), topical antiseptic agent (OR = 2.491, *p* < 0.005), non-surgical mechanical therapy (OR = 2.689, *p* < 0.001), surgical therapy (OR = 2.009, *p* < 0.01), and remove implant (OR = 3.486, *p* < 0.001) to treat peri-implant diseases, compared to NPDs. However, the years of practice didn’t show significant influence on treatment dentists would use, after adjusting other factors (*p* > 0.05, Table 3).

**Table 2:** Multiple logistic regression with GEE model to analyze the factors influencing whether the dentist would prefer to refer the patient to someone else for treatment or not when faced with peri-implant diseases

	OR	Confidence interval	*p* value
**Gender**			
Female (reference)	1	–	
Male	0.781	0.389 – 1.572	0.489
**Age**	0.980	0.922 – 1.042	0.521
**Years of practice**			
≤ 10 years (reference)	1	–	
> 10 years	0.839	0.256 – 2.745	0.771
**Location of occupation, N (%)**			
Local clinics	1	–	
Hospitals	0.890	0.329 – 2.405	0.819
Medical centers	1.524	0.711 – 3.265	0.278
**Specialty**			
NPD (reference)	1	–	
Periodontist	0.412	0.225 – 0.755	0.004*
**Resource of patient**			
Other doctor-treated (reference)	1	–	
Self-treated	0.010	0.005 – 0.020	< 0.001*
**Peri-implant disease**			
Peri-implant mucositis (reference)	1	–	
Peri-implantitis	1.860	1.318 – 2.625	< 0.001*

Abbreviations: *OR* Odds ratio, *NPD* Non-periodontist dentist

^*^: significant difference at *p* < 0.05

**Table 3:** Multiple logistic regression with GEE models to analyze the factors influencing the treatment modality for peri-implant disease by dental clinicians

OR	Regular follow	Occlusal adjustment	Oral hygiene instruction	Topical antiseptic agent	Topical antibody application	Systemic antibody application	Non-surgical mechanical therapy	Surgical therapy	Laser treatment	Remove implant
**Age**	0.973	1.021	1	1.002	0.965	0.992	1.008	1.040	0.965	0.996
**Gender**										
Female (reference)	-	-	-	-	-	-	-	-	-	-
Male	0.950	0.704	0.659	0.857	1.273	0.866	0.675	0.715	2.236	1.489
**Location of occupation**										
Local clinics (reference)	-	-	-	-	-	-	-	-	-	-
Hospitals	1.495	0.616	0.485	0.624	0.968	1.390	1.000	1.061	0.608	0.945
Medical centers	1.029	0.427^a^	0.579	1.275	0.754	0.963	0.877	0.859	0.533	0.529^a^
**Years of practice**										
≤ 10 years (reference)	-	-	-	-	-	-	-	-	-	-
> 10 years	0.661	0.821	0.678	0.572	1.605	1.707	0.687	0.953	2.695	1.331
**Specialty**				-						
NPD (reference)	-	-	-	-	-	-	-	-	-	-
Periodontist	0.965	2.283^b^	3.751^c^	2.491^c^	1.449	1.142	2.689^d^	2.009^b^	1.126	3.486^d^
**Resource of patient**				-						
Other doctor-treated (reference)	-	-	-	-	-	-	-	-	-	-
Self-treated	0.803	1.790^d^	1.037	1.169	1.321^a^	1.140	1.577^d^	2.075^d^	1.446^b^	1.641^d^
**Peri-implant disease**				-						
Peri-implant mucositis (reference)	-	-	-	-	-	-	-	-	-	-
Peri-implantitis	0.687^c^	1.474^d^	0.860	0.946	0.935	1.355c	0.851	5.240^d^	1.697^d^	4.255^d^

Abbreviations: *OR* Odds ratio, *NPD* Non-periodontist dentist

a, b, c, and d indicate statistical differences as *p* < 0.05, *p* < 0.01, *p* < 0.005, and *p* < 0.001, respectively

### Pursuit of knowledge

Almost all of periodontists and NPDs agreed that peri-implant diseases are associated with periodontitis, at 98.6% for periodontists and 97.9% for NPDs, without significant difference shown (*p* = 0.594). Moreover, both periodontists and NPDs are interested in learning more about basic periodontology and peri-implant diseases (*p* = 0.685, Table 4).

**Table 4:** Decisions made for Question V and Question VI by periodontists and non-periodontist dentists (NPDs)

	Yes		No		Total	*p*
	n	%	n	%	n	
Periodontists	72	98.6%	1	1.4%	73	0.594
NPDs	92	97.9%	2	2.1%	94	
Total	164	98.2%	3	1.8%	167	
**Question VI: Would you like to learn more knowledge about basic periodontology and peri-implant diseases?**						
Periodontists	72	98.6%	1	1.4%	73	0.685
NPDs	93	98.9%	1	1.1%	94	
Total	165	98.8%	2	1.2%	167	

Abbreviations: *NPD* Non-periodontist dentist

## Discussion

When subjects encounter an implant originally placed by another dentist that is diagnosed with peri-implant mucositis or peri-implantitis, he or she is more likely to refer the patient back to the original dentist at 72% and 83%, respectively (Fig. 1). However, when an implant placed by the dentist him/herself is diagnosed with peri-implant mucositis or peri-implantitis, the dentist would prefer to treat the disease him/herself at 92% and 89%, respectively. This demonstrated the concept that most dentists would like to treat peri-implant diseases if the implant were placed by him/herself and to refer peri-implant diseases to original dentists if the implant was placed by other dentists (Fig. 1 and Table 1). In other words, most participants agreed that the ideal dentist to treat peri-implant diseases would be the ones who placed the dental implant initially. This finding implies patients would return to the dentist who originally performed the implant therapy if any complications occurred, as a dentist unfamiliar with the patient’s original condition would refrain from treating the patient. Abrahamsson et al*.* investigated patients referred from other dentists for treatment of peri-implantitis in their study, and found that such patients usually demonstrated unrealistically high expectations [14]. Additionally, a study in United Kingdom showed that 78% of dentists considered the treatment outcomes of peri-implantitis unpredictable [20]. When a dentist encounters a patient with high expectations, but low predictability in treatment outcome, it would make sense for a dentist not to choose treating patients with peri-implantitis if implants were originally placed by another dentist.

Some NPDs in Question I to IV considered referring the patient to other periodontists (14.9%, 13.8%, 8.5%, and 11.7%, respectively) or oral surgeons (3.2%, 0%, 1.1%, and 1.1%, respectively). This showed that NPDs might believe periodontists or oral surgeons possess more capacity for performing implant procedures and managing peri-implant diseases [17]. When implants placed by dentists themselves become diagnosed with peri-implant diseases, a greater number of periodontists preferred to treat the patient themselves, compared to NPDs (Fig. 2C and D). Furthermore, periodontists were significantly more likely to treat the patients who come for treatment of peri-implant disease, by him/herself (OR = 0.412, *p* = 0.004, Table 2). This may be attributed to the fact that patients with a history of periodontitis are more likely to be accompanied with peri-implant diseases, and periodontists would thus be more accustomed to treating peri-implant diseases [21, 22]. In addition, most of the scientific journals about periodontology are also committed to publishing implant-related articles and improving education in periodontology and implant dentistry, so periodontists would have more access to the newest knowledge on implant dentistry and treatment procedures [23].

Compared to peri-implant mucositis, peri-implantitis results in not only inflammation around the soft tissue, but also progressive bone loss. Additionally, peri-implant mucositis may be successfully treated using non-surgical procedures if detected early, whereas peri-implantitis usually requires more invasive surgical treatment, etc. [8, 16]. Therefore, dentists would more likely to use variable procedures to treat peri-implantitis, such as regular follow, occlusal adjustment, systemic antibody application, surgical therapy, laser treatment, and remove implant (*p* < 0.05) (Table 3). These complex cases usually require more specific knowledge, broader training, and more in‐depth clinical experience to manage the patient’s condition in a sustainably successful manner [24]. Since NPDs hadn’t received more advanced periodontal and implant-related surgery before, they may choose to refer the patient to other specialists or use the non-invasive procedure to treat the patient (Tables 2 and 3). In fact, an ideal implant placement and maintenance of peri-implant health following implant placement should be equally essential for long-term success of the implant therapy [25, 26]. Similarly, successful management of peri-implant disease require dentists to be able to not only diagnose and treat the disease, but also make appropriate further referrals [14, 27]. Although many treatment procedures for peri-implant disease had been proposed and validated, the referral of peri-implant disease was not yet established well and explored, compared to the referral of periodontitis [10, 11, 28]. This study provided an overview for referral pattern and treatment modality among dentists when they were facing peri-implant disease, which could be a foundation in developing the rule for referral of peri-implant disease among dentists.

In our study, periodontists significantly preferred choosing series variable therapeutic approaches to achieve satisfactory results (Fig. 3A), including occlusal adjustment, oral hygiene instruction, topical antiseptic agent, non-surgical mechanical therapy, surgical therapy, and remove implant for the treatment of peri-implant diseases, compared to NPDs (Table 3, *p* < 0.05). However, the clinical years of practice didn’t influence the dentists’ treatment decision after adjusting all other factors (*p* > 0.05) (Table 3 and Fig. 4). These results indicated the significant differences of treatment modalities between periodontists and NPDs, and the periodontal specialty training could significantly affect the treatment modality of dental dentists, instead of clinical experience. This was similar to the finding of previous studies in other countries showing postgraduate specialty training significantly impacted clinical decision-making of dentists [29, 30]. Interestingly, the above mentioned treatment procedures were also considered as the most effective and useful therapeutic procedures to treat peri-implant diseases [31–33].

With the development of implant dentistry, it is expected that dentists will face a dramatically increased need to care for peri-implant diseases. The education for dentists is crucial and should include the responsibility of maintenance of implants, and handling of biological or technical complications [21, 34, 35]. Hence, the education of basic periodontal and peri-implant disease-related knowledge should be highlighted for any dentist practicing implant dentistry [36]. Despite the increased research focusing on implant dentistry, the management of peri-implant diseases still remains a challenge. The treatment of peri-implant diseases vary greatly between dentists with different backgrounds and trainings [37–41]. The time-consuming yet unpredictable long-term prognosis for management of peri-implant diseases was also not cost-effective for most dental practitioners [42]. Studies have demonstrated that the current implant education at the undergraduate or postgraduate levels didn’t instill confidence in general practitioners to provide and maintain dental implants [4, 43, 44]. This study shows that not only do periodontists express a desire to pursue continuing education in basic periodontology and peri-implant disease, but also NPDs (Table 4). According to Ng et al.’s study, more and more general dental practitioners (61%) practiced implant therapy by the year 2008 compared to 39% in 2004, so a strong demand for improving educational programs in implant dentistry is implicated [45, 46].

It is quite important to adequately use representative sample size to reflect the scientific value and true clinical situations. Considering the amount of sample size, we had conducted sample size calculation prior to the study to strengthen the power of analysis. Therefore, the total minimum sample was 128 dental practitioners, including at least 64 periodontists and 64 NPDs required to achieve the power of 80% and OR of 0.4. Moreover, the sensitivity and specificity of the study were assessed to verify the validity of the questionnaire. Hopefully, the process of determining the appropriate number of participants will be beneficial for drawing more realistic conclusions from our findings.

Concerning whether the general practitioners could diagnose per-implant mucositis or peri-implantitis clinically and fully comprehend all Questions are quite important, so the face-to-face interview and explanation of scenarios were performed to each participant, instead of e-mail, social media, or phone call. Moreover, all subjects had the experience of dental implant placement in patients and most of them would like to become further educated in basic periodontology and peri-implant diseases (Table 4). According to Fig. 2, when an implant placed by the dentist him/herself is diagnosed with peri-implant mucositis or peri-implantitis, most of subjects would prefer to treat the disease. Therefore, both periodontists and non-periodontal dentists in this study should be able to understand definition of the peri-implant mucositis or peri-implantitis clinically. The results of the study also showed that the severity of the disease affected the decision of referral and treatment procedure by subjects (Tables 2 and 3). This implied these subjects could sense the different severity of peri-implant mucositis or peri-implantitis, so they change their decisions for treatment modality and referral. This was also in line with previous studies which indicated different therapeutic methods for peri-implant mucosisit and peri-imlantitis [31–33].

In this study, we tried to recruit respondents who could stand for the most of periodontal specialists and general practitioners, and have experiences of performing dental implant placement. Therefore, the questionnaires were distributed to participants attending the conferences of three major academies in Taiwan: Academy of Family Dentistry (AFD), Taiwan Academy of Periodontology (TAP), and the Academy of Dental Implantology. The results of this study also showed a broad range of subjects of periodontists and non-periodontist dentists with different age, genders, years of practice, and location of occupation (Table S2). In recently years, various learning system, such as web-based learning and study clubs, has been prevalent, so many dentists enthusiastic about learning could also access the information of up-today knowledge and continue their education, instead of attending a physical conference. However, obviously, these individuals, who attended the physical conference, might be more enthusiastic about learning and more updated with latest trends in implant dentistry as compared to their counterparts who don't attend such events regularly. The result of this study still presented a trend that different scenarios and training backgrounds could affect the dentists’ treatment modality and referral pattern. The observed findings in this novel study was commendable and could be perceived as a panoramic view of the current conditions dentists must face on a daily basis. To establish a comprehensive care for patient with implant therapy, developing a rule for referral of peri-implant disease among dentists, treating the peri-implant disease early, and maintenance of peri-implant health should be highlighted. A further larger-scale study, including a broader geographic range with random sampling and providing more detailed clinical scenarios, would be needed and improved to obtain more comprehensive current situation generalized to the entire population of dentists in Taiwan [47, 48].

## Conclusion

Based on our finding, most dentists agreed that the ideal dentist to treat peri-implant diseases was the one who placed the dental implant initially. The periodontal specialty training, disease severity, and patient’s resource significantly influenced the referral pattern and treatment modality of dentists for treating an implant diagnosed with peri-implant disease. The importance of educating basic periodontal and peri-implant disease-related knowledge for all dentists regularly performing dental implant treatments was also highlighted.

## Supplementary Information

**Additional file 1. **Supplementary tables.

## Data Availability

The datasets of the current study are available from the corresponding author on reasonable request.

## Abbreviation

NPD: Non-periodontist dentists
