# Supplementary material for: The referral pattern and treatment modality for peri-implant disease between periodontists and non-periodontist dentists
Source: BMC Oral Health. 2023 Jun 27;23:427. doi: 10.1186/s12903-023-03135-3 (PMC10303747; doi:10.1186/s12903-023-03135-3)
Supplement: Supplementary file 1 — Additional file 1. Supplementary tables. [file 12903_2023_3135_MOESM1_ESM.doc]

**Supplemental Tables**

**Table S1 The references for the designed questionnaire**

| **Study** | **Method** | **Participants** | **Outcomes** | **Number of related factors** |
| --- | --- | --- | --- | --- |
| Kraatz et al. (2019) | Mail | 77 dentists | Periodontal diagnosis, treatment, and referral patterns | 12 |
| Lind et al. (2022) | Retrospective analysis of patient’s charts | 369 charts | Periodontal referral patterns | 8 |
| Chang et al. (2014) | Online responses | 1,200 dentists | How periodontists and other  dental professionals view the scope of periodontics | 13 |
| Lee et al. (2020) | Interview | 380 dentists | Dentists’ clinical decision-making | 7 |
| Lang-Hua et al. (2012) | Mail | 100 dentists | Attitudes towards implant dentistry | 14 |

**Table S2 Demographic of the study subjects**

|  |  | Periodontists |  | NPDs | Total | p |
| --- | --- | --- | --- | --- | --- | --- |
|  |  | N = 73 |  | N = 94 | N = 167 |  |
| **Age, Mean (SD)** |  | 37.25 (9.1) |  | 38.49 (13.3) | 37.95 (11.66) | 0.476 |
| **Gender, N (%)** |  |  |  |  |  | 0.041 |
| Male |  | 48 (65.8) |  | 75 (79.8) | 123 (73.7) |  |
| Female |  | 25 (34.2) |  | 19 (20.2) | 44 (26.3) |  |
| **Years of practice, N (%)** |  |  |  |  |  | 0.02 |
| 1~5 years |  | 13 (17.8) |  | 38 (40.4) | 51 (30.5) |  |
| 6~10 years |  | 30 (41.1) |  | 19 (20.2) | 49 (29.3) |  |
| 11~15 years |  | 13 (17.8) |  | 10 (10.6) | 23 (13.8) |  |
| >15 years |  | 17 (23.3) |  | 27 (28.7) | 44 (26.3) |  |
| **Location of occupation, N (%)** |  |  |  |  |  | 0.051 |
| Medical centers |  | 37 (50.7) |  | 44 (46.8) | 81 (48.5) |  |
| Hospitals |  | 14 (19.2) |  | 8 (8.5) | 22 (13.2) |  |
| Local clinics |  | 22 (30.1) |  | 42 (44.7) | 64 (38.3) |  |

Abbreviations: NPD, non-periodontist dentist
